# Relationship Between Cognitive Fusion, Experiential Avoidance, and Obsessive–Compulsive Symptoms in Patients With Obsessive–Compulsive Disorder

**DOI:** 10.3389/fpsyg.2021.655154

**Published:** 2021-04-12

**Authors:** Ai Xiong, Xiong Lai, Siliang Wu, Xin Yuan, Jun Tang, Jinyuan Chen, Yang Liu, Maorong Hu

**Affiliations:** Department of Psychosomatic Medicine, The First Affiliated Hospital of Nanchang University, Nanchang, China

**Keywords:** cognitive fusion, experiential avoidance, obsessive-compulsive disorder, depression, acceptance commitment therapy

## Abstract

**Objective:** This study aimed to explore the relationship among cognitive fusion, experiential avoidance, and obsessive–compulsive symptoms in patients with obsessive–compulsive disorder (OCD).

**Methods:** A total of 118 outpatient and inpatient patients with OCD and 109 healthy participants, gender- and age-matched, were selected using cognitive fusion questionnaire (CFQ), acceptance and action questionnaire−2nd edition (AAQ-II), Yale–Brown scale for obsessive–compulsive symptoms, Hamilton anxiety scale, and Hamilton depression scale for questionnaire testing and data analysis.

**Results:** The levels of cognitive fusion and experiential avoidance in the OCD group were significantly higher than those in the healthy control group (*P* < 0.05). Regression analysis results showed that, in predicting the total score of obsessive–compulsive symptoms, AAQ-II (*β* = 0.233, *P* < 0.05) and CFQ (*β* = 0.262, *P* < 0.01) entered the equation, which explained 17.1% variance. In predicting anxiety, only AAQ-II (*β* = 0.222, *P* < 0.05) entered the equation, which explained 13% variance. In the prediction of depression, AAQ-II (*β* = 0.412, *P* < 0.001) entered the equation, which explained 17.7% variance.

**Conclusion:** Cognitive fusion and experiential avoidance may be important factors for the maintenance of OCD, and experiential avoidance can positively predict the anxiety and depression of OCD patients.

## Introduction

Obsessive–compulsive disorder (OCD) is the fourth most common psychiatric disorder following depression, alcohol dependence, and phobia, with obsessions and compulsions as the main symptoms (De Putter and Koster, 2017). The lifetime prevalence of OCD is 1–3%, and the 12-month prevalence in China is 1.63% (Huang et al., 2019). OCD can hinder the quality of life and social function of individuals and bring serious damage to individuals and their families (Medeiros et al., 2017). It is one of the main clinical features of neurological diseases, which plays an important role in their pathogenesis. Studies have found that the incidence of OCD with comorbid anxiety (including generalized anxiety disorder and panic disorder) is 19.4%, and the incidence of anxiety with comorbid OCD is 8.8% (Yuan et al., 2001). Studies show that patients with OCD often have depressive symptoms, and depression is a common comorbidity of OCD, with a comorbidity rate of 50–60% (Overbeek et al., 2002; Besiroglu et al., 2007). Previous longitudinal studies have shown that the improvement of depressive symptoms can partially mediate the improvement of obsessive–compulsive symptoms; therefore, the study of anxiety and depressive symptoms in patients with OCD is necessary to explore the pathogenesis of OCD (Anholt et al., 2011; Zandberg et al., 2015).

However, the pathogenesis of OCD has not yet been accurately explained, and biological and psychological studies and interventions have shown the complexity of the etiology of OCD. Apart from genetic and neurobiological factors, social-psychological factors can also affect the occurrence and development of OCD. Previous imaging studies have suggested that OCD patients have abnormal neural circuits in the frontal lobe striatum and frontal lobe–parietal lobe–limbic system. The frontal parietal lobe is an important node in these two circuits, and its internal structure and functional connection with other brain regions can affect the occurrence of OCD (Gürsel et al., 2018). At present, research on the social-psychological factors of OCD primarily focuses on the personality and cognition dimensions. Perfectionism, as a cross-diagnostic process, drives various pathological processes (Group OCCW, 1997). The Compulsive Cognition Working Group (Group OCCW, 1997) noted that perfectionism was considered a risk factor for OCD development (Egan et al., 2011). Traditional cognitive dimension research and intervention primarily focus on the dysfunctional beliefs of patients, and the cognitive model of OCD points out that the dysfunctional beliefs play a key role in the pathogenesis of OCD (Sarawgi et al., 2013). However, with the rise of the third generation of cognitive–behavioral therapy represented by acceptance commitment therapy (ACT), research on the cognitive level has been expanded. The perspective no longer focuses on dysfunctional beliefs but focuses on patients' mental flexibility, acknowledges the universality of pain, requires patients to accept rather than resist pain and bad experience, and emphasizes the role of mindfulness (Dindo et al., 2017).

Cognitive fusion and experiential avoidance, as important theoretical models of acceptance and commitment therapy, are important factors for the formation and maintenance of many psychological disorders (Bohlmeijer et al., 2011; Afari et al., 2019; Woolf-King et al., 2019). Cognitive fusion indicates that patients equate thoughts with reality, and they are not aware of what he or she is thinking at the moment. Experiential avoidance refers to a patient's attempt to get rid of, avoid, or suppress unwanted personal experiences. Cognitive fusion and experiential avoidance correlates with “mutual deduction,” that is, they interact with each other and aggravate patients' negative psychological emotions (Hayes, 2004). When patients have pathological cognitive fusion and experiential avoidance, psychological flexibility is reduced, and psychological problems occur.

In an experiment that analyzes the mental flexibility of cancer patients and reported results of anxiety and depression, researchers found no significant differences in psychological flexibility based on gender (Hulbert-Williams and Storey, 2016). Gloster et al. (2011) assessed mental flexibility in clinical and non-clinical samples, and they found negligible associations between mental flexibility, gender, and age. However, the age of onset in OCD shows a bimodal pattern, with two peak ages of 12–14 years old and 20–22 years old, and the onset after 35 years old only accounts for 15% (Grant et al., 2007; de Mathis et al., 2008). Previous literature has defined the early onset and late onset of OCD by different classifications such as 15, 16, 17, 21, 30, and 50 years old, among which studies involving 15–18 years old are more common. Therefore, taking 18 years old as the node to study whether mental flexibility is different between patients with early onset and late onset has reference value for clinical psychotherapy. Studies have also found differences in the severity of symptoms such as examination and cleaning between male and female OCD patients (Cherian et al., 2014; Mathes et al., 2019). Therefore, investigating whether gender difference exists in the mental flexibility of OCD patients is important to study the relationship between obsessive–compulsive symptoms and mental flexibility.

Studies have found that cognitive fusion and experiential avoidance are significantly correlated with anxiety and depression (Hitchcock et al., 2018; Thomas and Bardeen, 2020). Acceptance commitment therapy reduces people's negative emotions by reducing experiential avoidance and increasing the acceptance of negative thoughts and emotions (Bohlmeijer et al., 2011). In addition, cognitive fusion can predict unacceptable obsessive thoughts (Reuman et al., 2016). The experiential avoidance of OCD patients can also predict their obsessive–compulsive symptoms, and the higher the level of experiential avoidance, the more serious the obsessive–compulsive symptoms (Wetterneck et al., 2014; Stockton et al., 2018). Therefore, whether improving mental flexibility can reduce the anxiety and depression of patients with OCD and improve the symptoms of OCD is the focus of this study.

### Present Study

Relevant studies have supported/demonstrated the efficacy of ACT in improving OCD symptoms by addressing cognitive fusion and experiential avoidance (Dehlin et al., 2013; Twohig et al., 2015). However, most studies on ACT only focus on the improvement of mental health and symptoms (Levin et al., 2017; Bai et al., 2020). In addition, research on the relationship between psychopathological models and symptom levels is lacking. Few in-depth studies on the relationship between clinical symptoms and cognitive fusion and experiential avoidance in OCD patients are found. Therefore, based on the theoretical basis of ACT, using Chinese OCD patients as samples, this study intends to explore the relationship among obsessive–compulsive symptoms, cognitive fusion, and experiential avoidance; find treatment methods to alleviate symptoms based on two aspects, namely, cognitive fusion and experiential avoidance; provide a specific direction for ACT intervention in clinical OCD; and promote the localized development of ACT.

### Hypotheses

(1) Demographic differences were found between cognitive fusion and experiential avoidance scores in the clinical group of OCD, age of onset, and gender.
(2) Cognitive fusion and experiential avoidance were significantly correlated with OCD, anxiety, and depression.
(3) Cognitive fusion and experiential avoidance positively predicted the level of OCD and the level of anxiety and depression in OCD patients.

## Methods

### Participants

#### The Experimental Group

Patients with OCD treated in the First Affiliated Hospital of Nanchang University from March 2018 to December 2019 were selected. The diagnosis of OCD was confirmed by a trained senior psychiatrist according to the Diagnostic and Statistical Manual of Mental Disorders Fifth Edition (DSM-5), and patients should meet the inclusion and exclusion criteria. The inclusion criteria were as follows: (1) OCD patients aged 13–45 years, first visit, and no history of psychological treatment, (2) volunteer to participate in this study, and (3) able to communicate with the researchers smoothly. The exclusion criteria were as follows: (1) patients with organic mental disorders, schizophrenia, and mood disorders, (2) patients with visual or auditory impairment, and (3) persons who were dependent on drugs or alcohol, pregnant, or lactating women. A total of 133 participants were recruited; 15 were excluded according to the abovementioned criteria, and 118 patients with OCD were enrolled after obtaining informed consent. Among the patients, 61 were males, 57 were females, 49 were under 18 years old, and 69 were 18 years old and above. A total of 85 patients with early-onset obsessive–compulsive disorder and 33 patients with late-onset OCD were included in the study.

#### The Healthy Control Group

The recruitment of the healthy control was primarily conducted through online questionnaires. The inclusion criteria were as follows: (1) age 13–45, (2) no previous or current organic brain diseases or major physical diseases, (3) no previous or current mental illness and relevant family history, and (4) with informed consent. A total of 109 normal participants were enrolled, including 58 males and 50 females. There were 43 under the age of 18, and 66 were 18 years old and above.

No significant difference in gender and age was found between the two groups (*P* > 0.05). The experiment was approved by the Ethics Committee of the First Affiliated Hospital of Nanchang University, and informed consent was obtained from all the participants or their legal guardians or first-degree relatives.

### Research Tools

#### Obsessive–Compulsive Symptoms

The Yale–Brown scale for OCD (Y-BOCS) consists of 10 items, in which the first 5 items assess obsessions, and the last 5 items assess compulsions, each on a five-point scale of 0–4, with an overall score of 40. The higher the score, the worse the symptoms will be. This scale is commonly used for the assessment of obsessive–compulsive symptoms worldwide. The Chinese version of Y-BOCS has acceptable reliability and validity (Xu and Zhang, 2006), good consistency among evaluators, intraclass correlation coefficient (ICC) of each item, and total scale score of ≥0.82. Retest reliability was acceptable, where the ICC of each item and total scale score was ≥0.75. Furthermore, Cronbach's alpha was 0.75.

#### Cognitive Fusion

Cognitive fusion questionnaire (CFQ), compiled by Gillanders in 2010, contains two dimensions, namely, cognitive fusion and cognitive defusion, examining the degree of cognitive fusion from the pros and cons. In this study, the Chinese version of the CFQ was adopted. After the original questionnaire was translated into Chinese by Zhu Zhuohong's team, the Chinese draft was formed through discussion and modification by the research group. Two English professionals were invited to translate the questionnaire into English, which was adjusted under the joint evaluation and discussion of psychological experts and finally translated into Chinese. The Chinese version of the CFQ contains nine items, and the seven-point scoring method is adopted. The higher the score of cognitive fusion, the higher the degree of cognitive fusion, and the higher the score of cognitive defusion dimension, the lower the degree of cognitive fusion. Moreover, the reliability and validity of the Chinese version of the CFQ in the Chinese population were verified. After testing, the internal consistency coefficient was 0.92, and the retargeting reliability was 0.67, showing good reliability and validity, which could be used in relevant studies on cognitive fusion in China (Zhang et al., 2014).

#### Experiential Avoidance

Acceptance and Action Questionnaire-−2nd edition (AAQ-II, Chinese edition) is a Chinese version of the English version compiled by Bond. After obtaining the authorization of Bond, two graduate students of psychology in the Institute of Psychology, Chinese Academy of Sciences, independently translated the scale and formed the first draft of the Chinese version of AAQ-II after discussion and modification. Then, the questionnaire was translated back into English by two English professionals. Finally, the original scale and translated scale were collected to compare the differences between the two versions. The final translated scale consisted of seven items and adopted the seven-point scoring method. The higher the score, the higher the level of experiential avoidance. The internal consistency coefficient of the scale was 0.88, and the retest reliability was 0.80 (Cao et al., 2013).

#### Anxiety

Hamilton Anxiety Scale (HAMA), developed in 1960, was clinically used to assess the severity of the participants' anxiety disorders. The scale had 14 items and a score of 5. The scale contained two factors, namely, somatic anxiety and mental anxiety. The higher the total score of the scale, the more serious the anxiety (Zimmerman et al., 2020).

#### Depression

Hamilton Depression Scale (HAMD), a 17-item version (Zimmerman et al., 2013), was adopted in this study, which could be divided into five factors, namely, anxiety/somatization, weight, cognitive impairment, block, and sleep disorder. A score of 0–4 indicated that the higher the score, the more severe the depressive mood.

### Procedure

The participants were confirmed by two psychiatrists on the basis of the DSM-5 diagnostic criteria. The participants and guardians of the juvenile were informed of the purpose of the study by a graduate student in psychology. After obtaining informed consent, the psychology graduate student evaluated the participants according to the items of Y-BOCs, HAMA, and HAMD. The psychology graduate student would guide the participants to fill out the general demographic questionnaire, AAQ-II, and CFQ to ensure that participants under the age of 18 could understand the project.

### Statistical Treatment

SPSS 25.0 statistical software was used for data analysis, and *t* test was used to compare significant differences in cognitive fusion and experiential avoidance among different genders and age of onset of OCD patients. Pearson correlation analysis was used to explore the significant correlation between the scores of OCD, anxiety, and depression and cognitive fusion and experiential avoidance. Finally, according to the results of correlation analysis, cognitive fusion and experiential avoidance were considered as independent variables to conduct multiple stepwise regression analysis on the levels of OCD, anxiety, and depression in patients with OCD and to investigate the predictive effect of cognitive fusion and experiential avoidance on the clinical symptoms of patients with OCD. *P* < 0.05 was considered statistically significant.

## Results

### Comparison of CFQ and AAQ-II Scores Between the OCD Group and Healthy Control Group

The cognitive fusion and experiential avoidance scores of the OCD group were significantly higher than those of the healthy control group (43.11 ± 8,677 *vs*. 31.1 ± 9.233, 34.92 ± 5.866 *vs*. 23.93 ± 6.823; *t* = 10.102, 13.05; *P* < 0.05; Table 1).

**Table 1:** Comparison of cognitive fusion and experiential avoidance scores between the obsessive–compulsive disorder (OCD) group and healthy control group.

**Variables**	**Group**	***N***	**Mean**	***S***	***t***
AAQ-II	OCD	118	34.92	5.866	13.05^***^
	Control	109	23.93	6.823	
CFQ	OCD	118	43.11	8.677	10.102^***^
	Control	109	31.1	9.233	

*AAQ-II, Acceptance and Action Questionnaire−2nd edition; CFQ, cognitive fusion questionnaire*.

^*^P < 0.05, ^**^P < 0.01,

*** *P < 0.001*.

### Scores of Experiential Avoidance and Cognitive Fusion Among Different Demographic Characteristics of OCD Patients

One-way ANOVA results showed no significant difference in cognitive fusion (*t* = −1.097, *P* = 0.275) and experiential avoidance (*t* = 1.208, *P* = 0.23) scores among patients with OCD at age of onset. In addition, the cognitive fusion and experiential avoidance scores (*t* = −1.358, *P* = 0.177; *t* = 0.083, *P* = 0.934) of OCD patients did not show significant differences in gender variables (Table 2).

**Table 2:** Comparison of the scores of experiential avoidance and cognitive fusion among different demographic characteristics of obsessive–compulsive disorder patients (scores, ‘*x* ± *s*).

**Variables**	***N***	**CFQ**	***t*/*F***	***P***	**AAQ-II**	***t*/*F***	***P***
Gender			−1.358	0.177		0.083	0.934
Male	61	42.07 ± 8.31			34.97 ± 5.44		
Female	57	44.23 ± 8.99			34.88 ± 6.34		
Age of onset			−1.097	0.275		1.208	0.23
Early onset	85	42.56 ± 7.80			35.33 ± 5.65		
Late onset	33	44.52 ± 10.22			33.88 ± 6.36		

*AAQ-II, Acceptance and Action Questionnaire-−2nd edition; CFQ, cognitive fusion questionnaire*.

### Correlation Analysis Results of Obsessive–Compulsive Symptoms, Anxiety, Depression, Cognitive Fusion, and Experiential Avoidance

The average scores of OCD patients were 43.11 ± 8.68 for cognitive fusion, 34.92 ± 5.87 for experiential avoidance, 24.16 ± 6.59 for obsessive–compulsive symptoms, 22.31 ± 5.05 for anxiety, and 20.36 ± 6.01 for depression. Pearson's product–moment correlations were used to calculate the relationship among cognitive fusion, experiential avoidance, obsessive–compulsive symptoms, anxiety, and depression. The results showed that cognitive fusion was positively correlated with obsessive–compulsive symptoms (*r* = 0.367, *P* < 0.001) and anxiety (*r* = 0.297, *P* < 0.01), and experiential avoidance was positively correlated with obsessive–compulsive symptoms (*r* = 0.351, *P* < 0.001), anxiety (*r* = 0.325, *P* < 0.001), and depression (*r* = 0.425, *P* < 0.001). However, no significant association was found between cognitive fusion and depression (Table 3).

**Table 3:** Correlation analysis results of obsessive–compulsive symptoms, anxiety, depression, cognitive fusion, and experiential avoidance (*n* = 118, *r*).

**Variables**	**CFQ**	**AAQ-II**	**Y-BOCS**	**HAMA**	**HAMD**
CFQ	1				
AAQ-II	0.488^***^	1			
Y-BOCS	0.367^***^	0.351^***^	1		
HAMA	0.297^**^	0.325^***^	0.148	1	
HAMD	0.025	0.425^***^	0.199^*^	0.622^***^	1

*AAQ-II, Acceptance and Action Questionnaire-−2nd edition; CFQ, cognitive fusion questionnaire; Y-BOCS, Yale–Brown Scale for obsessive–compulsive disorder; HAMA, Hamilton Anxiety Scale; HAMD, Hamilton Depression Scale*.

* *P < 0.05*,

** *P < 0.01*,

*** *P < 0.001*.

### Results of Regression Analysis of Obsessive–Compulsive Symptoms, Anxiety, and Depression

Multiple linear regression analysis was conducted with Y-BOCS total score of OCD patients as the dependent variable, age of onset and gender as control, and AAQ-II and CFQ score as predicting variables. When predicting the total score of obsessive–compulsive symptoms, AAQ-II (*β* = 0.233) and CFQ (*β* = 0.262) were calculated. The OCD patients' cognitive fusion level and experiential avoidance level could positively predict the severity of OCD and its joint, which explained 17.1% variance, respectively. In addition, multiple linear regression analysis was conducted with OCD patients' anxiety score as the dependent variable, age of onset and gender as control, and AAQ-II and CFQ score as prediction variables. In predicting anxiety, cognitive fusion has been excluded, and only AAQ-II (*β* = 0.222) was included in the equation. Experiential avoidance significantly predicted and influenced the level of anxiety in patients with OCD, which explained 13% variance. In predicting depression, cognitive fusion showed no significant correlation with depression; thus, unary linear regression was performed using the experiential avoidance level as the predictor variable and age of onset and gender as control. Based on the results of AAQ-II (*β* = 0.412), the experiential avoidance level showed a significantly predicted effect for depression, which explained 17.7% variance. The variance inflation factor of each regression equation was not greater than the evaluation index value of 5, indicating no linear coincidence (multicollinearity) among the independent variables entering the regression equation (Table 4).

**Table 4:** Results of regression analysis of obsessive–compulsive symptoms, anxiety, and depression.

**Dependent variables**	**Predictor variables**	***B***	**SE**	***t***	***t***	**Adjusted *R*^**2**^**
Y-BOCS	AAQ-II	0.262	0.11	0.233	2.376^*^	0.171
	CFQ	0.199	0.075	0.262	2.653^**^	
HAMA	AAQ-II	0.191	0.087	0.222	2.207^*^	0.130
HAMD	AAQ-II	0.422	0.086	0.412	4.887^***^	0.177

*AAQ-II, Acceptance and Action Questionnaire-−2nd edition; CFQ, cognitive fusion questionnaire; Y-BOCS, Yale–Brown Scale for obsessive–compulsive disorder; HAMA: Hamilton Anxiety Scale; HAMD: Hamilton Depression Scale*.

* *P < 0.05*,

** *P < 0.01*,

*** *P < 0.001*.

## Discussion

The results of this study showed no significant differences in cognitive fusion and experiential avoidance scores of OCD patients with regard to gender and age of onset, which is in agreement with previous relevant research (Gloster et al., 2011; Hulbert-Williams and Storey, 2016).

The results of the correlation analysis of cognitive fusion and obsessive–compulsive symptom's level showed no significant positive correlation between cognitive fusion and obsessive–compulsive symptoms, which was consistent with the results of Wells and Papageorgiou (1998). A significant positive correlation was also found between the scores of experiential avoidance and obsessive–compulsive symptoms. The AAQ-II entries showed the avoidance and control of compulsive thoughts and emotions, which might aggravate patients' compulsive thoughts and negative emotions, thereby trapping them in thoughts and restricting them to take effective actions to improve the status. The association between AAQ-II content and obsessive–compulsive symptoms explains the positive correlation. In addition, cognitive fusion and experiential avoidance also showed a positive correlation with anxiety scores, but experiential avoidance was positively correlated with the depression level, whereas cognitive fusion was not significantly correlated with the depression level. This result was inconsistent with previous research results. Bardeen and Fergus (2016) studied the interaction between cognitive fusion and experiential avoidance on anxiety, depression, stress, and post-traumatic stress in a large community group and found a correlation between cognitive fusion and depression level probably because the content of the cognitive fusion of OCD patients did not point to self-derogatory thoughts, and the process of cognitive fusion was a thinking process, which was not consistent with the thinking retardation shown by depression.

In the multiple linear regression analysis of obsessive–compulsive symptoms' total score, cognitive fusion was included in the regression equation, which was consistent with the meta-cognitive model of OCD proposed by Wells and Papageorgiou (1998) and the cognitive model of OCD proposed by Reuman et al. (2016). Both models showed that thought fusion belief was the main cause of OCD. Reuman et al. (2016) first investigated the causal relationship between cognitive fusion and obsessional symptoms and found that cognitive fusion could predict unacceptable obsessive thoughts in obsessive symptoms. As a result of pathological cognitive fusion, the patient had confusion between thoughts and reality, and the patient was immersed in negative thoughts and emotions. Under the influence of negative thoughts, a patient's sense of self-efficacy decreased, deviated from the self-value, and finally led to OCD. Brain imaging studies of OCD have found that OCD patients had abnormal activity in the frontal lobe, parietal lobe, and other brain regions, resulting in impaired cognitive inhibition ability, compulsive ideas, and behavior (Grützmann et al., 2016; Zielinska et al., 2016). When patients have pathological cognitive fusion, they cannot get rid of their thoughts, which will lead to or aggravate the OCD. In addition, the experiential avoidance score was included in the regression equation, and the experiential avoidance could positively predict the level of obsessive–compulsive symptoms. Considering that the mechanism of experiential avoidance could relieve tension and reduce the negative experience brought by the threat situation, people often used experiential avoidance to avoid unwanted experience. The avoidance and control of negative emotions and adverse experiences in OCD patients will aggravate their psychological pain, lead to poor coping strategies, and aggravate the degree of compulsive symptoms, which are consistent with relevant research results (Wetterneck et al., 2014; Stockton et al., 2018). The higher the level of experiential avoidance, the lower the psychological flexibility, and the more likely psychological problems will occur. The effective treatment of OCD by ACT also showed that reducing the level of experiential avoidance played an important role in the improvement of OCD (Rohani et al., 2018).

AAQ-II was included in the regression equation for the regression analysis of anxiety in OCD patients. Experiential avoidance can significantly and positively predict anxiety of OCD patients. When patients were immersed in thoughts and eager to escape from such a situation, they would suffer from intense anxiety caused by a sense of control. Experiential avoidance tended to increase the frequency and degree of negative emotions (Rochefort et al., 2018). The effect of experiential avoidance on anxiety was consistent with relevant research results (Wicksell et al., 2008). In the regression equation of depression, cognitive fusion was not considered as a predictive variable because no correlation between cognitive fusion and depression was found, and only AAQ-II was included in the regression equation. Experiential avoidance could positively predict the depressive mood in patients with OCD, which was consistent with relevant research results (Hitchcock et al., 2018). The abovementioned results also suggested that the anxiety, depression, and psychological pain of OCD patients could be reduced by decreasing their experiential avoidance level (Bohlmeijer et al., 2011).

Although this study studied the relationship among cognitive fusion, experiential avoidance, and obsessive–compulsive symptoms in OCD patients, found that cognitive fusion and experiential avoidance had a positive predictive effect on the symptoms of OCD, and supported the effectiveness and practicality of ACT in treating OCD, lower levels of cognitive fusion and experiential avoidance improve the mental flexibility and clinical symptoms of OCD patients. This study has also some limitations: (1) The sample size included in this study is not large enough, and all of them are from the same hospital. Thus, the representativeness of the sample will be limited to a certain extent; (2) Given the cross-sectional nature of this study, inferences about the causality of the relationship remain limited. Longitudinal studies will help clarify this issue; (3) The pathological model of ACT includes cognitive fusion, experiential avoidance, conceptualized self, lack of value clarification, limited self-cognition, and invalid action six plates. This study only examines the cognitive fusion, experiential avoidance, and obsessive–compulsive symptoms. Moreover, the ACT pathological model should be further improved. (4) Various dimensions of obsessive–compulsive symptoms, such as the symmetry/counting/ordering and taboo thoughts/checking subtypes, and different symptom subtypes showed different neuropsychological defects, which might have different etiologies (Bragdon et al., 2018). However, this study did not classify the specific symptom dimensions. In the future, based on the predictive effect of cognitive fusion and experiential avoidance on the degree of obsessive–compulsive symptoms in this study, the relationship among cognitive fusion, experiential avoidance, and different dimensions of obsessive–compulsive symptoms could be considered to study patients with different subtypes of OCD and improve the results of this study.

## Conclusions

The study findings indicated that the cognitive fusion and experiential avoidance of OCD patients might be important factors for the maintenance of OCD, and experiential avoidance could positively predict the anxiety and depression of OCD patients.

## Data Availability Statement

The raw data supporting the conclusions of this article will be made available by the authors, without undue reservation.

## Ethics Statement

The studies involving human participants were reviewed and approved by the ethics committee of The First Affiliated Hospital of Nanchang University. Written informed consent to participate in this study was provided by the participants, and where necessary, the participants' legal guardian/next of kin.

## Author Contributions

AX: responsible for data analysis and writing articles; MH: responsible for revising the article: XL, SW, XY, JT, JC, and YL: responsible for collecting data. All authors contributed to the article and approved the submitted version.

## Conflict of Interest

The authors declare that the research was conducted in the absence of any commercial or financial relationships that could be construed as a potential conflict of interest.
